# Association between Low Forced Vital Capacity and High Pneumonia Mortality, and Impact of Muscle Power

**DOI:** 10.3390/jcm12093272

**Published:** 2023-05-04

**Authors:** Nanako Shiokawa, Tatsuma Okazaki, Yoshimi Suzukamo, Midori Miyatake, Mana Kogure, Naoki Nakaya, Atsushi Hozawa, Satoru Ebihara, Shin-Ichi Izumi

**Affiliations:** 1 Department of Physical Medicine and Rehabilitation, Tohoku University Graduate School of Medicine, Sendai 980-8575, Japan; 2 Center for Dysphagia, Tohoku University Hospital, Sendai 980-8574, Japan; 3 Department of Preventive Medicine and Epidemiology, Tohoku Medical Megabank Organization, Sendai 980-8575, Japan; 4 Department of Internal Medicine and Rehabilitation Science, Tohoku University Graduate School of Medicine, Sendai 980-8574, Japan; 5 Department of Physical Medicine and Rehabilitation, Tohoku University Graduate School of Biomedical Engineering, Sendai 980-8575, Japan

**Keywords:** forced vital capacity, muscle strength, older people, pneumonia mortality, sarcopenia, cohort study

## Abstract

Impaired % predicted value forced vital capacity (% FVC) is related to higher all-cause mortality in aged adults, and strong muscle force may improve this relationship. A muscle disease, sarcopenia, causes higher mortality. We aimed to identify the unknown disease that relates impaired % FVC with higher mortality in aged adults among the three major leading causes of death, and the effect of strong leg force on this relationship. Cox proportional hazard model analyzed the longitudinal Tsurugaya cohort that registered 1048 aged Japanese for 11 years. The primary outcome was the relationship between % FVC and mortality by cancer, cardiovascular disease, or pneumonia. Exposure variables were % FVC or leg force divided by 80% or median values, respectively. The secondary outcome was the effects of leg force on the relationship. Among the diseases, % FVC < 80% was related only to higher pneumonia mortality (hazard ratio [HR], 4.09; 95% CI, 1.90–8.83) relative to the % FVC ≥ 80% group before adjustment. Adding the leg force as an explanatory variable reduced the HR to 3.34 (1.54–7.25). Weak leg force might indicate sarcopenia, and its prevention may improve higher pneumonia mortality risk related to impaired % FVC, which we may advise people in clinical settings.

## 1. Introduction

Major indicators of respiratory functions include forced vital capacity (FVC) and % predicted value FVC (% FVC). Previous studies showed that impaired FVC/% FVC were related to higher mortality in the general population [1,2,3,4,5]. In general, impaired FVC/% FVC are major indices for the diagnosis of interstitial lung disease, which is accompanied by higher mortality [6,7]. To exclude potential interstitial lung disease patients, two previous studies excluded people who complained about respiratory symptoms and showed that impaired FVC/% FVC were related to higher mortality [8,9].

Previous studies have reported that extremity muscle weakness was related to higher mortality [10,11,12,13,14,15]. There is a moderate correlation between the extremity and respiratory muscle strengths. In addition, there is a moderate correlation between FVC/% FVC and respiratory muscle power [16,17,18,19]. In aged adults, the development of pneumonia was related to respiratory muscle weakness [20]. Moreover, the possibility of a relationship between pneumonia-induced death and respiratory muscle weakness was suggested [20]. In aged males, strong leg force showed a potential to improve the relationship between impaired % FVC and higher mortality [9]. 

Currently, the disease that causes the relationship between higher mortality and impaired FVC/% FVC in the general population is unknown. Moreover, the effect of strong muscle force on the relationship between the disease-induced higher mortality and impaired FVC/% FVC is unknown. 

The primary objective of this research was the identification of the disease that causes the relationship between higher mortality and impaired % FVC in aged adults. Next, this study aimed to identify the impact of strong leg force on the relationship between the disease-induced higher mortality and impaired % FVC. 

The longitudinal cohort, the Tsurugaya project, was analyzed to identify the suggested objectives. We first evaluated a relationship between the impaired % FVC and the three major leading causes of death in Japan. The three major leading causes of death were cancer, cardiovascular disease, and pneumonia. We hypothesized the relationship between impaired % FVC and higher pneumonia mortality and the beneficial effects of the strong leg force on this relationship.

## 2. Materials and Methods

### 2.1. Participants

In 2002, the Tsurugaya project enrolled older adults aged ≥70 years and conducted comprehensive geriatric assessments [9]. The baseline data were collected by the survey performed in the assessments. We obtained informed consent from all participants involved in the study. 

### 2.2. Examinations

We examined lung function using a spirometer (OST 80A, Chest Co., Tokyo, Japan). We took the best result among the 3 trials. We calculated the % FVC values based on participants’ gender, age, and height [21]. We performed the first measurements in 2002 in accordance with the recommendation of the American Thoracic Society [22]. For the evaluation of FVC, we applied reference values announced by the Japanese Respiratory Society (JRS) in 2001. In addition, as a cut-off value for % FVC, we used 80%, announced by JRS in 2001. JRS announced the present reference values for Japanese in 2014; however, to be consistent with other Tsurugaya cohort studies, the reference values published in 2001 were applied in this study [23]. As for the leg force (w/kg), we evaluated its extension force using Combi Anaeropress3500 (Tokyo, Japan), a horizontal leg force measurement device [24]. The leg force was measured 5 times, and the average of the 2 strongest leg forces was calculated. We divided the average by the body weight [24]. We divided the participants into strong and weak leg force groups according to the median values of the gender-dependent leg force: ≥13.0 w/kg for males and ≥7.3 w/kg for females in the strong group. To evaluate dyspnea, we asked the participants to inspire through an external circuit. The external circuit was set with 3 steps of resistive load (cmH_2_O/L/s); the lowest load was 10, the middle was 20, and the highest was 30. We asked them to report their feeling using the modified Borg scale. The modified Borg scale categorizes dyspnea on a scale from 0 to 10. The number 0 is scaled as no dyspnea, and the number 10 is the greatest dyspnea. As baseline breathing, the participants were asked to breathe without resistive load for 1 min. We excluded participants who selected 2 or greater at the baseline breathing as potential interstitial lung disease patients in the sensitivity analyses [9]. The questionnaire survey collected sociodemographic and medical information. Date of birth, gender, past medical history (pneumonia, malignant disease, heart disease, stroke, diabetes mellitus, and hypertension), smoking status, and medications with statins and angiotensin-converting enzyme (ACE) inhibitors were included in the questionnaire. We listed ACE inhibitors because they improve cough and swallowing reflexes and prevent the onset of pneumonia [25]. The smoking status was a categorical variable; we categorized the smoking status of participants into current, past, or never. We evaluated symptoms of depression using the Geriatric Depression Scale (GDS) written in Japanese with a 30-point scale [26], and examined cognitive function using the Mini–Mental State Examination (MMSE) written in Japanese [9]. Serum samples were isolated without asking for fasting, and a clinical testing laboratory assessed the serum albumin and total cholesterol concentrations.

### 2.3. Mortality Follow-Up

The primary endpoint was death from pneumonia, cancer, and cardiovascular disease. Causes of death were classified in accordance with the International Classification of Disease, 10th Revision (ICD10). Deaths from pneumonia were classified as J12–18 and 69; from cancer, deaths were classified as C0–26, 30–41, 43–58, 60–97; from cardiovascular diseases, deaths were classified as I20–28, 30–52, 60–89, and 95–99.

We obtained data regarding death from the Sendai Municipal Authority. We surveyed the cause of death by checking hospital records or the data submitted to the Japan arteriosclerosis longitudinal study coordinating center. The Japan arteriosclerosis longitudinal study involved 21 cohort studies in Japan and included the Tsurugaya project [27]. We followed the participants from 30 March 2003 to 1 July 2012.

### 2.4. Statistical Analysis 

% FVC divided participants into 2 groups, and their characteristics at baseline were compared; chi-square tests compared categorical variables, and unpaired *t*-tests compared continuous variables. To evaluate the cumulative survival rate, we compared 2 groups divided by the % FVC. The Kaplan–Meier method and log-rank tests were used for the comparison. We calculated the hazard ratios (HRs) and 95% confidence intervals (CIs) of the mortality in this study using the Cox proportional hazard model. We set the reference group as the participants with % FVC ≥ 80%. Then, 2 models were ran to assess the correlation between the 2 groups for % FVC and mortality. Model 1 was defined as a model without adjustment. Gender and age were adjusted in Model 2. We also adjusted Model 2 with smoking. We divided the participants into 2 groups by the median values of the leg force. As the secondary outcome, we evaluated the impact of muscle force on the relationship between pneumonia mortality and % FVC divided into 2 groups. Model 1 was defined as a model without adjustment. In Model 2, we added leg force as an explanatory variable. Age and gender were adjusted in Model 3. Sensitivity analyses were performed for the evaluation of the robustness. The evaluated robustness was the relationship between mortality by pneumonia and % FVC divided into 2 groups. Statistical examinations were conducted using the software IBM SPSS Statistics 24.0 (International Business Machines Corporation, Armonk, NY, USA). We used the post hoc power analysis to evaluate the power of the main result. The software Power and Precision 4.1 (Biostat, Englewood, NJ, USA) was used for the evaluation. We interpreted *p* < 0.05 as statistically significant.

## 3. Results

**Figure 1:**
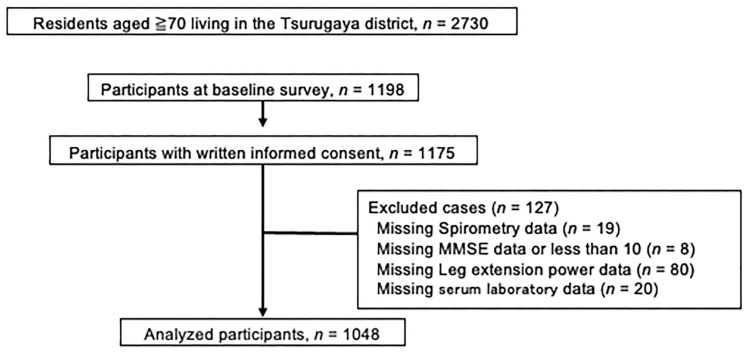
A schematic diagram outlining the enrollment in this study. MMSE = Mini–Mental State Examination.

In 2002, we recruited participants for the Tsurugaya project and invited all aged residents aged 70 years and older (*n* = 2730). At the baseline survey, 1198 participants were enrolled in the project. We obtained informed consent from 1175 participants. The study flow chart was shown in Figure 1. We excluded 19 participants without spirometry data. To keep the measured indices reliable, we used the Mini–Mental State Examination (MMSE). Participants missing the MMSE score or <10 were excluded (*n* = 8). Participants missing or with incomplete leg force measurement records were excluded (*n* = 80). Participants without serum laboratory data were also excluded (*n* = 20). Finally, we analyzed 1048 participants.

To divide the participants into two groups by % FVC, we used its clinical cut-off value of 80%. The characteristics of the baseline survey are shown in Table 1. Males occupied 42.2% of the participants, and 75.7 (4.8) years of age, average (standard deviation [SD]), was the average age of the participants. Between the two groups, we found significant differences in gender, history of suffering from pneumonia, MMSE, current smoking status, leg extension force, total cholesterol level, and serum albumin level. However, the differences in the MMSE scores were small: 26.7 (3.4) in % FVC < 80% and 27.4 (2.6) in ≥80%. The albumin levels showed a similar trend: 4.34 (0.3) in % FVC < 80% and 4.30 (0.3) in ≥80%.

We previously showed the relationship between higher all-cause mortality and impaired % FVC in the Tsurugaya cohort [9]. Next, we determined the disease that caused the relationship between higher mortality and impaired FVC/% FVC among the three major leading causes of death in Japan. We analyzed the data registered in 2002 and followed until 2012. During the 8310 person years of follow-up, there were 57 deaths from cancer (251 person years), 38 deaths from cardiovascular diseases (180 person years), and 26 deaths from pneumonia (129 person years). We analyzed the relationship between mortality, and the % FVC was divided into ≥80% and <80% in each disease (Table 2). We set the reference group as the participants with % FVC ≥ 80%. Model 1 was defined as a model without adjustment. Gender and age were adjusted in Model 2. Among the pneumonia group, % FVC < 80% was related to higher mortality; the HR (95% CIs) was 4.09 (1.90 to 8.83) in Model 1 and 3.08 (1.41 to 6.71) in Model 2. In cancer and cardiovascular disease groups, % FVC < 80% was not related to higher mortality in either Model 1 or 2; the HRs were 1.08 (0.57 to 2.05) in cancer and 1.88 (0.95 to 3.74) in cardiovascular disease groups in Model 1. The adjustment for smoking in Model 2 did not essentially change the results; the HRs were 2.91 (1.33 to 6.33) in pneumonia, 0.87 (0.46 to 1.66) in cancer, and 1.57 (0.78 to 3.15) in cardiovascular disease groups. The power of the HRs of the pneumonia mortality was 0.97 with the total duration, hazard rates, and attrition rates; the sample size was 825 in the % FVC group ≥ 80%, and 223 in the % FVC group < 80%, and alpha (0.05, 1-tail).

Next, sensitivity analyses of the relationship between % FVC and mortality were performed. Generally, interstitial lung disease patients are associated with higher mortality with impaired % FVC. Dyspnea is one of the typical symptoms of interstitial lung disease. Therefore, we evaluated dyspnea. Accordingly, we excluded 21 participants that had the potential to suffer from interstitial lung disease. The results did not essentially change after the exclusion; the HR was 3.75 (1.59 to 8.82) in Model 1 (Table A1). In general, aged pneumonia patients reach death by repeating the onset of pneumonia [17]. Thus, we excluded participants with a past history of pneumonia and conducted a sensitivity analysis. The results were somewhat confusing; the HR was 3.07 (1.31 to 7.18, *p* = 0.010) in Model 1 and 2.31 (0.98 to 5.45, *p* = 0.056) in Model 2 (Table A2).

**Figure 2:**
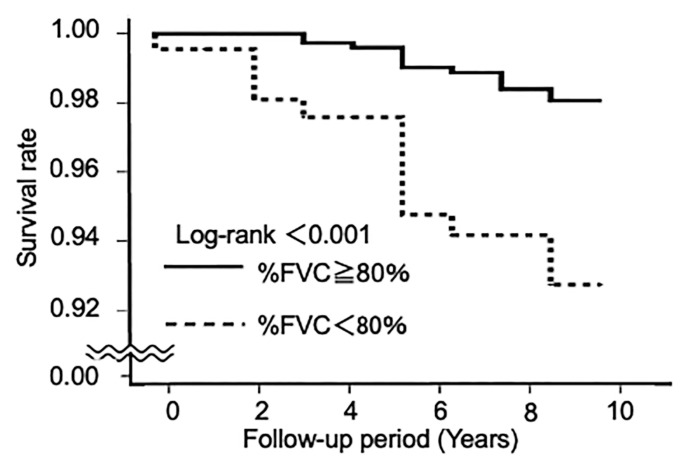
Kaplan–Meier survival curves showing the cumulative survival rates according to the FVC% predicted.

The number of deaths caused by pneumonia was 13 (1.6%) out of the 825 participants in the % FVC ≥ 80% group and 13 (5.8%) out of the 223 participants in the % FVC < 80% group. Figure 2 shows Kaplan–Meier survival curves associated with death caused by pneumonia according to the FVC% predicted. The % FVC < 80% group showed a significantly lower cumulative survival rate than the % FVC ≥ 80% group (log-rank test, *p* < 0.001).

We next evaluated the impact of muscle force on the relationship between % FVC and pneumonia mortality. Model 1 was a univariate model of this relationship (Table 3). The participants were divided into two groups, strong and weak, by the gender-dependent median values of the leg force. We defined the reference group as the participants with % FVC ≥ 80% and a strong leg force group. We added the leg force as an explanatory variable to the % FVC in Model 1 and showed it in Model 2. In the % FVC < 80% group, we observed a reduction in the HR 3.34 (1.54 to 7.25) in Model 2, whereas it was 4.09 in Model 1. The leg force in Model 2, shown in Table 3, was an unadjusted model, and the weak leg force was related to higher mortality; the HR was 5.27 (1.81 to 15.41), which we interpreted as independent from % FVC. Model 3 was further adjusted for age and gender. The HR was 2.59 (1.18 to 5.68) for the % FVC < 80% group. An adjustment of Model 3 to smoking did not essentially change the results; the HR was 2.50 (1.14 to 5.47) for the % FVC < 80% group.

## 4. Discussion 

Among the three major leading causes of death, impaired % FVC was related to higher pneumonia mortality in community-dwelling aged adults. Strong leg force may beneficially affect this relationship. 

The follow-up period of this study was from 2003 to 2012. The four leading causes of death were cancer, heart disease, pneumonia, and cerebrovascular disease during this period in Japan. However, the number of deaths by cerebrovascular disease was 15, and was insufficient for analysis. Therefore, we gathered death by heart and cerebrovascular diseases and analyzed them as cardiovascular diseases. A previous study analyzed 1265 aged adults with chronic obstructive pulmonary disease, asthma, or other diseases and reported cause-specific mortality rates of % FVC < 80% [1]. They recruited participants between January 1996 to July 1999 and followed until 30 January 2002. They reported higher mortality rates with pulmonary and cerebrovascular diseases. In contrast to their study, our study did not recruit participants with specific diseases and carried out a long-term follow-up. 

Respiratory function and extremity muscle strength have moderate correlations with respiratory muscle strength [16,17,18,19]. Respiratory muscle force regulates the effectiveness of coughing. The cough clears the airways and plays a central role in pneumonia protection [28]. Thus, strong leg force might beneficially affect the mortality risk of pneumonia via its correlation with respiratory muscle force and effective coughing. 

Generally, sarcopenia is an aging-related muscle dysfunction defined by muscle weakness, low muscle mass, and performance [29]. Muscle weakness comes to the forefront among these indices [30]. A previous study showed that weak respiratory muscle force and low muscle mass were risk factors for the onset of pneumonia [20]. These data may link sarcopenia to the respiratory muscles. Sarcopenia is crucial because it is related to higher mortality [31,32]. This study showed that the impaired % FVC was related to higher pneumonia mortality with the possible involvement of the leg force. When we take the above links together, this study may support the following idea; sarcopenia in respiratory muscles causes respiratory-related diseases, such as pneumonia, due to ineffective coughing and is possibly connected to death.

Interstitial lung disease patients show impaired % FVC and higher mortality [6]. Their common respiratory symptoms are dyspnea and coughing. This study examined dyspnea and attempted to exclude the participants that had the potential to suffer from interstitial lung disease. However, they might not have symptoms or impaired pulmonary functions. Thus, it is difficult to entirely exclude the participants with the potential to suffer from interstitial lung disease in the sensitivity analysis. The prevalence of interstitial lung disease patients was reported as approximately 6.3–76.0 cases per 100,000 people [7]. This study excluded 21 participants among 1048. Therefore, in addition to interstitial lung disease patients, non-interstitial lung disease patients might be excluded from the sensitivity analysis. 

We previously reported the beneficial effect of the strong leg force on the relationship between all-cause mortality and impaired % FVC in males but not in females [9]. Since pneumonia caused only six deaths in females in this study, we could not divide the participants by gender for further analysis. However, the strong leg force improved the relationship after adjustment for gender. Thus, other diseases than pneumonia might cause gender-dependent beneficial effects of the strong leg force on the relationship, or this may be a limitation of the current study. In addition, from the point of view of medical care costs, the costs were inversely associated with physical activity in aged adults [24]. The strength of the leg force partially represents physical activity [24]. Thus, strengthening the leg force and improving physical activity might have the potential of preventing pneumonia in addition to reducing medical care costs.

This study has some limitations. First, the participants were Japanese/East Asians, and the ethnicity of the sample was limited. Therefore, we may apply our results to Asian populations, but not to other ethnicities. Second, the number of pneumonia deaths was insufficient for numerous-factor adjustments. Third, we could not analyze data on the immune system, which plays essential roles in multiple diseases, especially in infectious diseases such as pneumonia [25,33,34]. Fourth, after excluding the past history of pneumonia in the sensitivity analysis, we encountered difficulties in the interpretation of the discrepancy of the HRs between Models 1 and 2. One possible explanation was that we could not know when the pneumonia developed, i.e., in youth or old age.

This study characterized the specific disease as pneumonia, which causes the relationship between impaired % FVC and higher mortality in community-dwelling aged adults. Preventing weak leg force may reduce mortality risk. Since we currently have few management strategies to improve FVC, we may suggest the potential benefits of strengthening muscle force to community-dwelling aged adults with impaired % FVC. 

## Figures and Tables

**Table 1:** The participants were divided by % FVC, and their baseline characteristics are shown.

Characteristics	Overall		% FVC				*p*-Value *
			<80%		≥80%		
**Number of Participants**	**1048**		**223**		**825**		
Age, mean (SD)	75.7	(4.8)	76.2	(4.5)	75.5	(4.9)	0.086
Men, *n* (%)	442	(42.2)	132	(59.2)	310	(37.6)	<0.001
Medical history, *n* (%)							
Pneumonia	100	(9.5)	35	(15.7)	65	(7.9)	<0.001
Cancer	70	(6.7)	12	(5.4)	58	(5.5)	0.451
Cardiovascular disease	161	(15.4)	34	(15.2)	127	(15.4)	0.957
Diabetes mellitus	146	(13.9)	36	(16.1)	110	(13.3)	0.278
Hypertension	392	(37.4)	92	(41.3)	300	(36.4)	0.180
Current smoking, *n* (%)	137	(13.4)	36	(16.4)	101	(12.5)	<0.001
MMSE, mean (SD)	27.3	(3.4)	26.7	(3.4)	27.4	(2.6)	0.048
Depressive symptoms, mean (SD)	9.0	(5.5)	9.3	(5.4)	8.9	(5.5)	0.356
Taking statins, *n* (%)	168	(16.0)	31	(13.9)	137	(16.6)	0.356
Taking ACE inhibitors, *n* (%)	78	(7.4)	17	(7.6)	61	(7.4)	0.886
Strong leg power ^†^, *n* (%)	529	(50.5)	86	(38.6)	443	(53.7)	<0.001
Weak leg power ^‡^, *n* (%)	519	(49.5)	137	(61.4)	382	(46.3)	
Total cholesterol (mg/dL), mean (SD)	203.8	(33.3)	199.1	(35.7)	205.1	(32.6)	0.018
Albumin (g/dL), mean (SD)	4.3	(0.3)	4.34	(0.3)	4.30	(0.3)	0.043

% FVC = % predicted value forced vital capacity, SD = standard deviation, MMSE = Mini–Mental State Examination, ACE = angiotensin-converting enzyme, * Continuous variables were evaluated by unpaired *t*-test, and the chi-squared test evaluated proportion variables to obtain the data. ^†^ Strong leg power; leg extension force ≥13.0 w/kg (male), ≥7.3 w/kg (female). ^‡^ weak leg power; leg extension force <13.0 w/kg (male), <7.3 w/kg (female).

**Table 2:** Relationship between % FVC divided into 2 groups and mortality by diseases.

	% FVC ≥ 80% (*n* = 825)	% FVC < 80% (*n* = 223)
**Pneumonia death** **(n)**	13	13
person years	129	
Model 1	1.00 (Reference)	4.09 (1.90–8.83)
Model 2	1.00 (Reference)	3.08 (1.41–6.71)
**Cancer death** **(n)**	45	12
person years	251	
Model 1	1.00 (Reference)	1.08 (0.57–2.05)
Model 2	1.00 (Reference)	0.87 (0.46–1.66)
**CVD death** **(n)**	26	12
person years	180	
Model 1 ^‡^	1.00 (Reference)	1.88 (0.95–3.74)
Model 2 ^§^	1.00 (Reference)	1.61 (0.80–3.21)

Hazard ratio (95% confidence interval), % FVC = % predicted value forced vital capacity, ^‡^ Model 1: defined as a model without adjustment, ^§^ Model 2: sex and age were adjusted, CVD = cardiovascular diseases.

**Table 3:** The muscle force affected the relationship between mortality by pneumonia and % FVC divided by 80%.

	% FVC ≥ 80% Strong Leg Power ^‡^	% FVC < 80% Weak Leg Power ^§^	*p*-Value
	Reference	HR (95% CI)	
**Model 1**			
FVC% predicted	1.00	4.09 (1.90–8.83)	<0.001
**Model 2**			
FVC% predicted Leg power	1.00	3.34 (1.54–7.25) 5.27 (1.81–15.41)	0.002 0.003
**Model 3**			
FVC% predicted Leg power	1.00	2.59 (1.18–5.68) 4.79 (1.59–14.45)	0.017 0.005

% FVC = % predicted value forced vital capacity, HR (95% CI) = hazard ratio (95% confidence interval), Model 1: defined as a model without adjustment, Model 2: leg power was split into 2 groups by the gender-dependent median values and added to Model 1, Model 3: sex and age were adjusted in Model 2, ^‡^ strong leg power; leg extension force ≥13.0 kg/w (male), ≥7.3 kg/w (female), ^§^ weak leg power; leg extension force <13.0 kg/w (male), <7.3 kg/w (female).

## Data Availability

The data that support the findings of this study are available from A.H. upon reasonable request.

## Appendix A

**Table A1:** The association between % FVC and pneumonia mortality after excluding potential interstitial lung disease patients.

	% FVC ≥ 80%	% FVC < 80%	*p*-Value
	Reference	HR (95%CI)	
**Model 1**	1.00	3.75 (1.59–8.82)	0.003
**Model 2**	1.00	2.93 (1.23–6.98)	0.016

% FVC = % predicted value forced vital capacity, HR (95% CI) = hazard ratio (95% confidence interval), Model 1: defined as a model without adjustment, Model 2: sex and age were adjusted.

**Table A2:** The relationship between % FVC and pneumonia mortality after the exclusion of participants with a past history of pneumonia.

	% FVC ≥ 80%	% FVC < 80%	*p*-Value
	Reference	HR (95%CI)	
**Model 1**	1.00	3.07 (1.31–7.18)	0.010
**Model 2**	1.00	2.31 (0.98–5.45)	0.056

%FVC = % predicted value forced vital capacity, HR (95% CI) = hazard ratio (95% confidence interval), Model 1: defined as a model without adjustment, Model 2: sex and age were adjusted.
